# Supervised machine learning approaches for early detection of metabolic and udder health disorders in dairy cows using sensor-derived data

**DOI:** 10.3389/fvets.2025.1726719

**Published:** 2025-11-19

**Authors:** Akvilė Girdauskaitė, Samanta Grigė, Karina Džermeikaitė, Justina Krištolaitytė, Dovilė Malašauskienė, Mindaugas Televičius, Greta Šertvytytė, Gabija Lembovičiūtė, Ramūnas Antanaitis

**Affiliations:** Large Animal Clinic, Veterinary Academy, Lithuanian University of Health Sciences, Kaunas, Lithuania

**Keywords:** dairy cows, machine learning, early lactation, innovative technologies, precision livestock farming

## Abstract

This study assessed five supervised machine learning (ML) models. Automated devices that continuously captured milk composition and behavioral data were used to monitor 206 Holstein cows from two commercial dairy farms. Milk yield, fat, protein, lactose, fat-to-protein ratio (FPR), somatic cell count (SCC), rumination time (RT), and body temperature were among the parameters that were noted. Cows were categorized as clinically healthy (*n* = 45), subclinical ketosis (*n* = 91), subclinical mastitis (*n* = 28), or clinical mastitis (*n* = 42) based on clinical examination, blood *β*-hydroxybutyrate (BHB) concentration, and milk indicators. Random Forest achieved the highest classification accuracy (0.857), followed by Gradient Boosting and Logistic Regression (0.833), while Decision Tree and Multilayer Perceptron reached 0.810. Compared to clinically healthy cows (4.45 ± 0.54%; 477.0 ± 36.0 min/day), subclinical ketosis cows had a greater milk fat content (5.21 ± 0.72%) and a shorter RT (336.9 ± 94.2 min/day). In comparison to clinically healthy cows (64.0 × 10^3^ cells/mL; 4.63 ± 0.16%), cows with clinical mastitis showed significantly greater SCC (416.8 × 10^3^ cells/mL) and lower lactose levels (4.56 ± 0.24%). These results demonstrate that integrating sensor-derived milk and behavioral data with ML algorithms enables early, non-invasive detection of health disorders, supporting proactive herd management.

## 1 Introduction

Dairy cows often face a negative energy balance (NEB) during the first 100 days of lactation due to the gap between nutrient intake and energy demand for milk production (1). Excessive lipomobilization to meet these energy needs increases circulating non-esterified fatty acids (NEFA), predisposing cows to metabolic disorders such as ketosis (2, 3). The physiological stress and immune suppression associated with NEB increase susceptibility to mastitis, and cows with higher lipomobilization are more likely to develop clinical cases (4). These metabolic disorders impair individual health, herd productivity, and overall management efficiency, emphasizing the need for nutritional adjustments and metabolic monitoring (5).

By delivering real-time data on milk composition, rumination behavior, and SCC, autonomous sensors have transformed dairy farming and allowed for quicker decisions that enhance productivity and welfare (6). Fat, protein, lactose, and total solids can all be measured by automated milking systems (AMS) that have in-line sensors. According to research, these sensors can reliably forecast the composition and quality of milk, allowing farmers to enhance milk quality and optimize feeding practices (7). For example, ongoing monitoring of fat and protein intake enables customized dietary modifications that can optimize milk production and avoid metabolic problems linked to inadequate nutrition. Continuous data gathering makes it possible to identify metabolic or udder health problems early on, enabling prompt interventions that lower veterinary expenses and increase herd longevity (6).

Accelerometers are used by rumination monitoring systems to continuously monitor chewing action and record jaw motions. Cows at risk for metabolic or udder health issues can be identified by reduced rumination duration, which frequently signals discomfort or illness (8). Producers can regularly evaluate udder health and take preventative measures thanks to automatic sensors that assess SCC in real-time. Because it is correlated with milk quality and yield, a high SCC indicates infection or inflammation in the udder and has major economic ramifications (9). Research has demonstrated that autonomous sensors can efficiently monitor SCC before clinical symptoms appear, enabling prompt actions and possibly lowering the prevalence of clinical mastitis within the herd (6). As a result, this method improves milk quality and total herd productivity in addition to improving animal wellbeing.

Particularly during the crucial transition period in dairy cows, ML models have demonstrated significant potential for the early diagnosis of both clinical and subclinical ketosis. According to recent studies, ML algorithms may recognize subclinical ketosis-typical behaviors, improving early detection when paired with automated monitoring systems (10, 11). Furthermore, a more thorough evaluation of dairy cow health is made possible by combining sensor-derived data with machine learning algorithms. ML models have the potential to automate the risk assessment of subclinical ketosis with high classification accuracy, converting complex datasets into useful insights for herd management, according to recent studies utilizing sensor-based techniques that incorporate physiological and management data (12). In order to predict mastitis in dairy cows, the integration of sensor data into multivariate dynamic models has also been investigated. This has shown that autonomously collected data can significantly reduce labor requirements for health monitoring while increasing the speed and accuracy of illness detection (13). The effectiveness and precision of these prediction models will continue to develop as research advances, providing dairy producers with strong instruments to guarantee the wellbeing and production of their herds (14).

Therefore, it was hypothesized that by analyzing real-time physiological, behavioral, and milk composition data, automated monitoring systems in conjunction with supervised ML algorithms could successfully identify early-stage metabolic and inflammatory disorders—such as subclinical ketosis and mastitis—in dairy cows during early lactation. This study used sensor-derived data from the Brolis HerdLine, Lely, and SmaXtec systems to assess how well several ML models classified dairy cows into health-related groups. The FPR, RT, SCC, milk temperature, and milk yield were among these integrated datasets. Additionally, during the first 100 days of lactation, the study sought to evaluate the classification performance of five supervised machine learning algorithms: Decision Tree, Logistic Regression, Multilayer Perceptron, Random Forest, and Gradient Boosting in identifying early metabolic and udder health imbalances in cows.

## 2 Materials and methods

### 2.1 Animal housing conditions of this study

The investigation was carried out on two commercial dairy farms close to Kaunas in central Lithuania. The time frame for the study was 15 March to 30 September 2025. Initially, 206 Holstein cows (127 cows from Farm A and 79 from Farm B) in early lactation (≤ 100 days in milk) were enrolled, comprising 138 multiparous and 68 primiparous cows. Cows were kept in loose-housing conditions in free-stall barns with rubber-matted cubicles and regulated airflow. Both farms offered unlimited access to clean drinking water and employed automated milking equipment (DeLaval or Lely Astronaut®). Welfare requirements were followed in maintaining environmental elements like temperature, humidity, and air flow. Throughout the year, cows were fed a total mixed ration (TMR) designed to meet or surpass the nutritional needs of a 550–650 kg Holstein cow that produced around 35 kg of milk every day. The TMR’s dry matter content was roughly 30–31% corn silage, 10% grass silage, 4% grass hay, 49–50% grain concentrate, and 6% mineral supplement. With an energy density of 1.60 Mcal/kg of net energy for lactation (NEL), the chemical composition averaged 48–51% dry matter (DM), 28–29% neutral detergent fiber (NDF), 18–20% acid detergent fiber (ADF), 38–39% non-fiber carbohydrates (NFC), and 15.8% crude protein (CP). Minor variations in TMR composition between the two farms reflected local differences in feed ingredient availability, as indicated by the presented percentage ranges. Although the base ration was formulated for cows producing approximately 35 kg of milk per day, concentrate supplementation was individually adjusted according to milk yield. Cows with higher milk production received 1–3 kg of additional concentrate per day through the robotic milking system.

Feeding took place twice a day, at roughly 7:00–8:00 and 15:00–16:00. To provide consistent feed access, automatic feed pushers redistributed the ration multiple times a day. The robotic milking system provided an extra 2 kg of concentrate every day to the cows. The cows’ average live weight was between 550 and 650 kg. The average annual energy-corrected milk yield per cow in 2024 was between 10,300 and 11,900 kg, with average protein and fat levels of 3.4–3.6% and 4.1–4.2%, respectively.

The State Food and Veterinary Service of the Republic of Lithuania authorized all experimental operations, which were carried out in compliance with institutional and national criteria for animal welfare (permission No. 135834789, 7 March 2025).

### 2.2 Parameter registration

All cows underwent continuous monitoring of eight parameters: milk yield, fat, protein, lactose contents, FPR, milk temperature, RT and SCC. The robotic milking systems employed by both farms had different kinds of in-line sensors. Farm A used the Lely Astronaut® system (Lely Industries N.V., Maassluis, The Netherlands), which included neck-mounted activity and rumination collars for all cows. Farm B used the DeLaval system integrated with the Brolis HerdLine in-line milk analyzer (Brolis Sensor Technology, Vilnius, Lithuania) and SmaXtec intrareticular boluses (SmaXtec Animal Care GmbH, Graz, Austria) to continuously record rumination activity. Thus, all cows were monitored using automated sensor systems, although the specific devices differed between farms.

Farm B recorded milk yield, temperature, and composition (fat, protein, lactose, and FPR) in real time using the Brolis HerdLine in-line milk analyzer. Throughout the milking process, the gadget measures milk flow every 5 s using a GaSb-based, tunable external cavity laser spectrometer that operates in the 2,100–2,400 nm spectral range. Each milking session’s final day results were calculated using flow-weighted averages.

Farm A’s Lely Astronaut® system evaluated SCC, RT, milk yield, and milk temperature automatically. Neck-mounted Lely collars recorded physical activity and rumination behavior, while in-line sensors integrated into the robotic milking apparatus measured milk temperature and SCC (represented as ×10^3^ cells/mL) for each milking.

In Farm B, SmaXtec intrareticular boluses were used to continuously record rumination activity. Each bolus was activated, linked to an individual cow ID, and administered orally by a trained veterinarian using a dedicated applicator.

### 2.3 Groups creation

This study focused on the early lactation period (≤ 100 days postpartum), during which cows experience major metabolic and physiological adjustments. Before sensor data analysis, all animals underwent a clinical examination and blood sampling to exclude cows with clinical metabolic disorders. BHB concentrations were measured using a portable meter (FreeStyle Optium Neo, Abbott Laboratories, UK) to confirm the absence of subclinical ketosis (BHB < 1.2 mmol/L). The final classification of animals into health-related groups was based on sensor-derived indicators (FPR, SCC, and RT) representing energy balance and udder health status. Throughout the study period, the same veterinarian conducted daily clinical examinations at 9:00 a.m. to evaluate each animal’s health and metabolic state. Concurrently, the Brolis HerdLine in-line milk analyzer and the Lely monitoring system were used to continually capture sensor-based data on milk yield, composition, and behavioral markers. The somatic cell count, rumination time, and fat-to-protein ratio were the main health indicators utilized for classification.

All cows were categorized into one of four health-related categories based on these factors, which represented energy balance and udder health status:

Group 1 (clinically healthy; *n* = 45) consists of cows with low SCC (<100 × 10^3^/mL), regular rumination activity, and normal FPR (1.20–1.39); Group 2 (subclinical ketosis risk; *n* = 91) consists of cows with shortened RT and higher FPR (≥1.40), which are signs of early-stage ketosis or negative energy balance; Group 3 (subclinical mastitis; *n* = 28) comprises cows with elevated SCC (>200 × 10^3^/mL) but normal FPR (1.20–1.39); Group 4 (clinical mastitis; *n* = 42) consists of cows that exhibit inconsistent rumination behavior and significantly high SCC (>400 × 10^3^/mL), which are indicative of severe udder inflammation.

**Figure 1:**
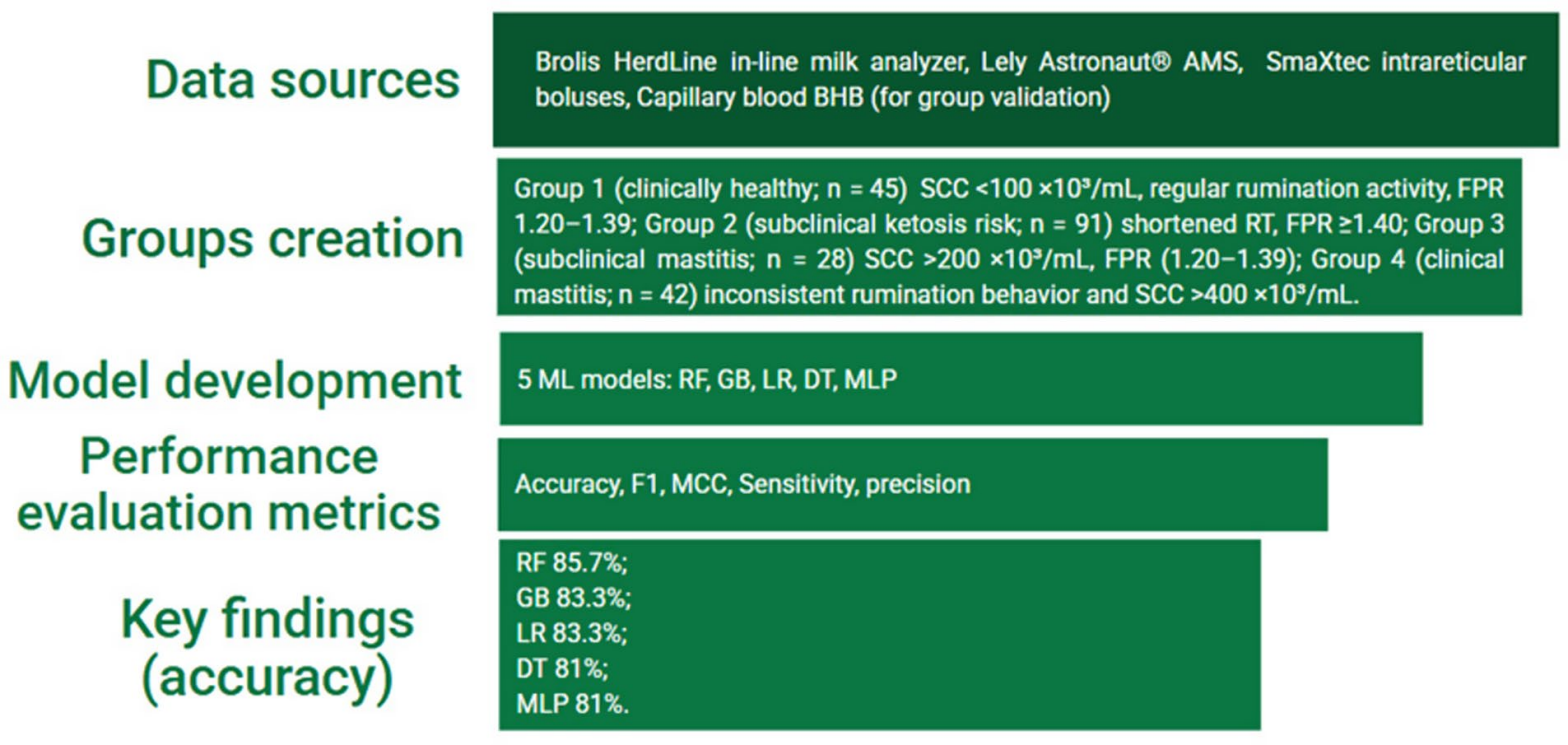
Study design and contributions.

The final study only included cows with complete information from the Lely and Brolis systems. Animals with incomplete sensor or analyzer data or evidence of systemic disease (such as lameness, metritis, or digestive problems) were eliminated. All monitored cows, however, fulfilled the requirements for inclusion and were kept under observation throughout the duration of the study. An overview of the study design and machine learning workflow is presented in Figure 1.

### 2.4 Predictive modeling workflow

All records were initially exported to Microsoft Excel (Microsoft, 2021) and subsequently imported into the KNIME analytics platform (version 5.4.4; KNIME GmbH, Konstanz, Germany) for analysis. All datasets were subjected to standard preprocessing prior to model training. Quality control tests were used to find and eliminate missing or implausible values. Categorical variables were numerically encoded to make them compatible with machine learning methods, while continuous variables were normalized to provide equal weighting among features. Boxplot visualization and descriptive statistics were used to analyze outliers. In order to reduce redundancy and enhance model performance, features were chosen using correlation analysis and variable relevance. Prior to processing, all datasets were carefully inspected to ensure completeness and accuracy.

To prepare the data for model training and validation, two consecutive partitioning steps were performed using the KNIME Partitioning node. In the first step, the dataset was divided into a training set (80%) and a testing set (20%) using relative sampling with the “draw randomly” option selected. In the second step, the training subset was further partitioned internally to enable cross-validation of the models. In both partitioning steps, a fixed random seed was applied to guarantee reproducibility of the sampling process across all analytical runs. Following data partitioning, a total of 164 cows were retained for analysis. The animals were allocated into four groups: Group 1 (*n* = 35), Group 2 (*n* = 75), Group 3 (*n* = 20), and Group 4 (*n* = 34). Five supervised ML algorithms were implemented to classify and predict metabolic conditions in early-lactation dairy cows: Decision Tree, Logistic Regression, Multilayer Perceptron, Random Forest, and Gradient Boosting. The DT model served as a transparent, rule-based classifier capable of identifying key predictive features through hierarchical data partitioning. The LR model provided a baseline linear approach for estimating the probability of class membership based on predictor variables. The MLP, a feedforward artificial neural network, was used to capture nonlinear relationships among features through iterative weight optimization. The RF model, composed of an ensemble of DT, was designed to enhance predictive performance and minimize overfitting; its maximum tree depth was limited to 10, and the number of trees was fixed at 100. The GB model, a sequential ensemble of weak learners, was applied to improve classification accuracy by iteratively minimizing residual errors from prior models.

### 2.5 Model evaluation criteria

For each machine learning model, a confusion matrix was generated to evaluate classification performance. The matrices summarized the correspondence between predicted and actual group memberships, allowing calculation of key performance metrics, including the number of correctly and incorrectly classified cases, overall accuracy (%), error rate (%), and Cohen’s Kappa coefficient (*κ*). Accuracy was calculated as the proportion of correctly.

classified instances out of the total number of cases, whereas error represented the percentage of misclassified records. Cohen’s Kappa was computed as a chance-corrected measure of agreement between predicted and observed classifications. The summarized results of all models are presented in Table 1.

**Table 1:** Summary of confusion matrix–based classification results for all model.

Model	Correctly classified (n)	Misclassified (n)	Accuracy (%)	Error (%)	Cohen’s Kappa (*κ*)
Decision tree	34	8	80.95	19.05	0.74
Random forest	36	6	85.71	14.29	0.81
Logistic regression	35	7	83.33	16.67	0.77
Gradient boosting	35	7	83.33	16.67	0.74
Multi-layer perceptron	34	8	80.95	19.05	0.74

### 2.6 Statistical and correlation analysis

All statistical analyses were carried out using the KNIME Analytics Platform. Descriptive statistics were computed for all physiological and milk composition variables. Normality was assessed using the Shapiro–Wilk test; when the assumption of equal variances was violated (Levene’s test, *p* < 0.05), Welch ANOVA followed by the Games–Howell *post hoc* test was applied.

Group comparisons were performed using one-way analysis of variance (ANOVA). When the assumption of homogeneity of variances was not met, the Games–Howell post hoc test was applied for pairwise group comparisons. Statistical significance was established at *p* < 0.05. Pearson correlation analysis was used to examine linear relationships among milk composition traits, physiological indicators, and production parameters. The magnitude and direction of correlations were interpreted according to established biological relevance.

## 3 Results

### 3.1 Model performance comparison

**Figure 2:**
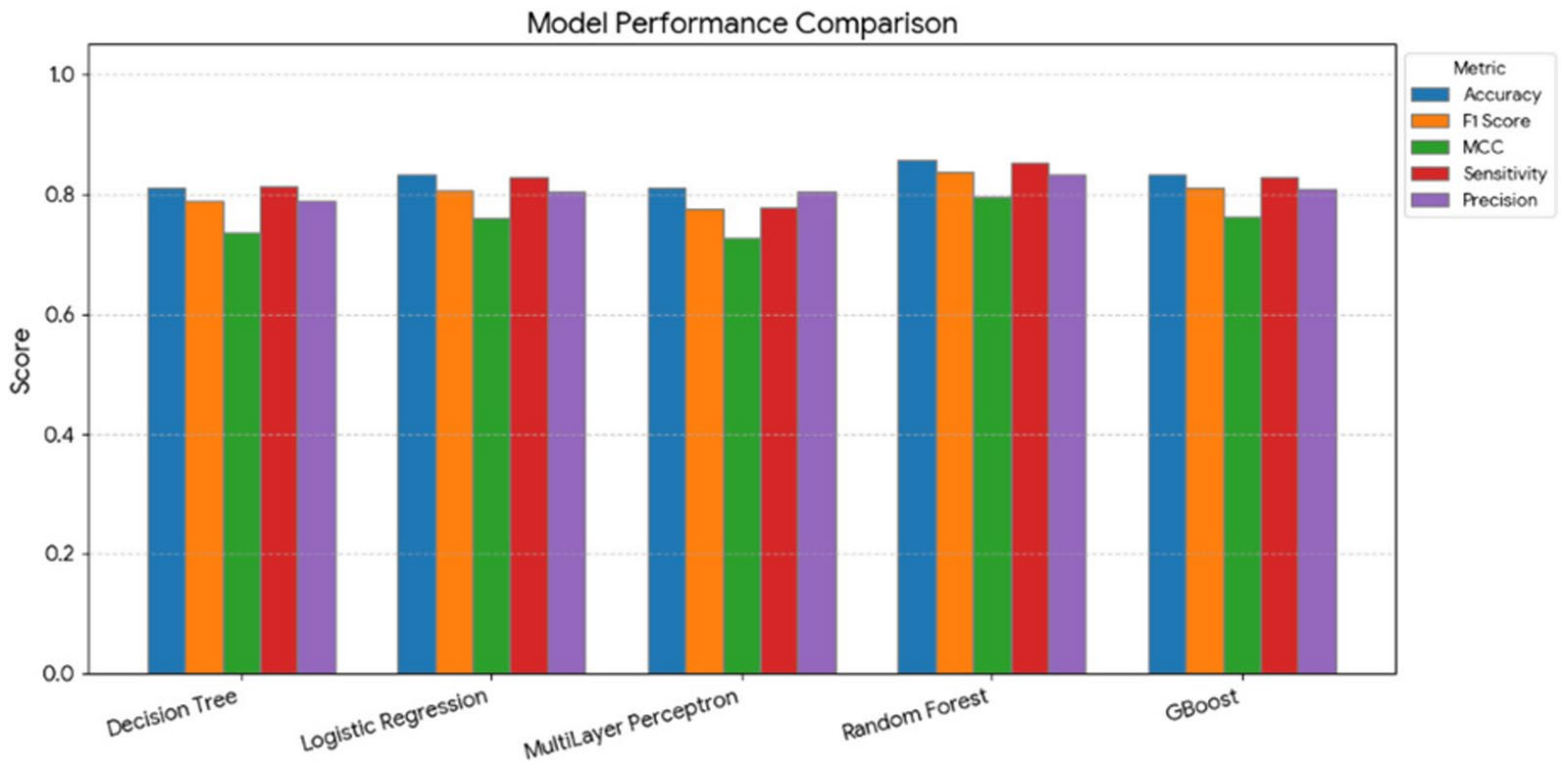
Comparison of Decision Tree, Logistic Regression, Multilayer Perceptron (MLP), Random Forest, and Gradient Boosted (GBoost) models in predicting dairy cow health categories (subclinical mastitis, healthy, subclinical ketosis, clinical mastitis). The figure displays Accuracy, Precision, Sensitivity, F1 score, and Matthews correlation coefficient (MCC). Ensemble models (Random Forest and GBoost) achieved the best overall performance.

The classification performance of five supervised machine learning models—DT, LR, MLP, RF, and GB—was compared for distinguishing four health categories: clinically healthy, subclinical ketosis, subclinical mastitis, and clinical mastitis. Overall, the models achieved high predictive accuracy, as summarized in Figure 2.

The RF model demonstrated the best overall predictive performance, achieving an accuracy of 0.857, precision of 1.000, sensitivity of 0.938, F1 score of 0.968, and MCC of 0.950. The GB model also performed strongly, with accuracy of 0.833, precision of 0.938, sensitivity of 0.938, F1 score of 0.938, and MCC of 0.833. Similarly, the LR model achieved a high accuracy of 0.833, precision of 1.000, sensitivity of 0.938, F1 score of 0.968, and MCC of 0.950, indicating a robust and well-calibrated classification performance.

In contrast, the DT model produced an overall accuracy of 0.810, precision of 0.714, sensitivity of 0.500, F1 score of 0.588, and MCC of 0.500, showing lower discriminative power compared with ensemble and regression-based methods. The MLP displayed moderate yet consistent results (accuracy = 0.810, precision = 0.875, sensitivity = 0.778, F1 score = 0.824, MCC = 0.781).

**Figure 3:**
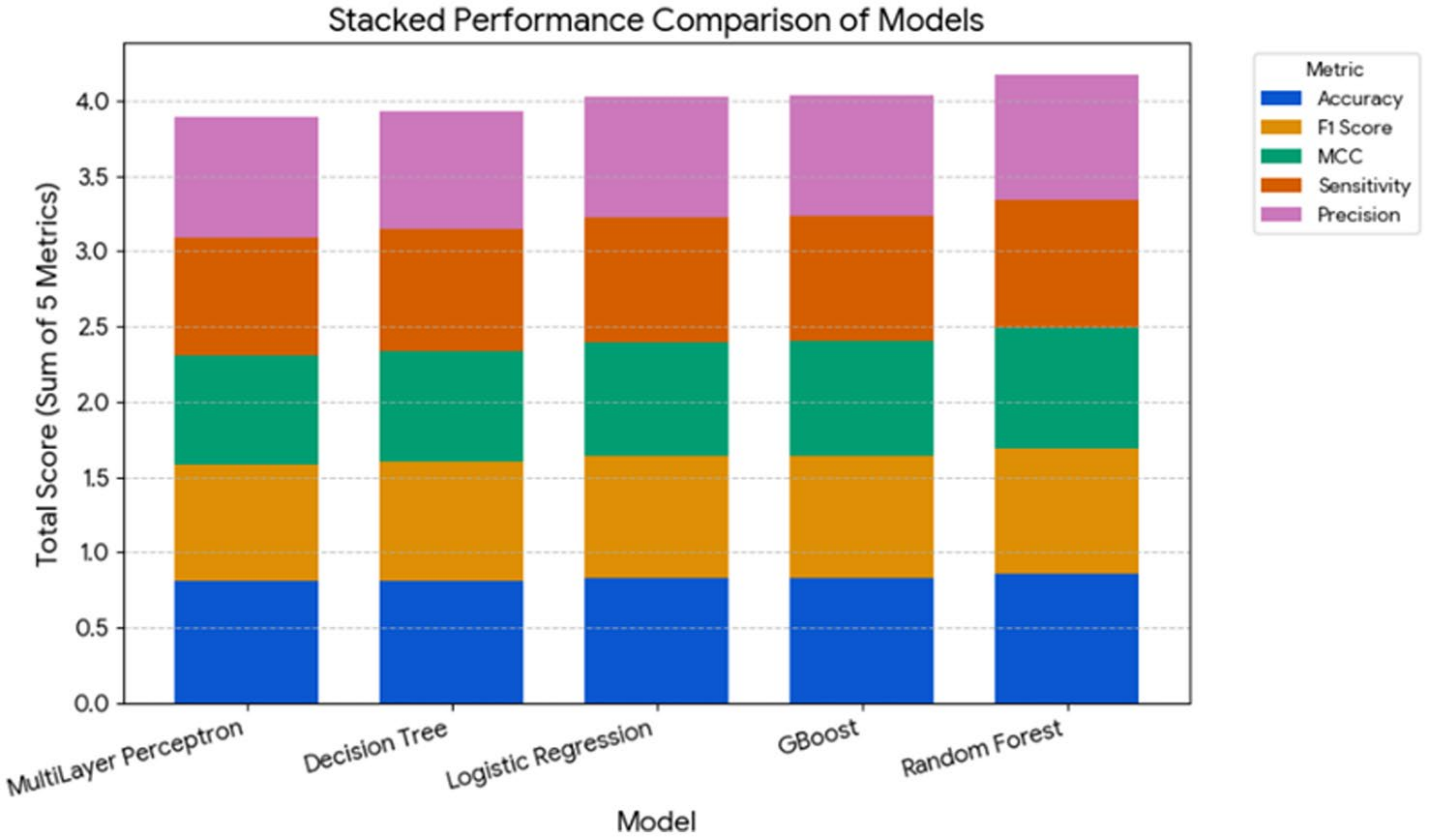
Stacked comparison of cumulative performance metrics across machine learning models for disease prediction in dairy cows. The stacked bar chart illustrates the cumulative performance of five machine learning models—MLP, Decision Tree, Logistic Regression, Gradient Boosted (GBoost), and Random Forest**—**used to classify cows into four health categories: (1) subclinical mastitis, (2) clinically healthy, (3) subclinical ketosis, and (4) clinical mastitis. The total height of each bar represents the sum of five performance metrics: accuracy, F1 score, MCC, sensitivity, and precision.

A comparative analysis of total model performance across the five metrics (Figure 3) revealed that ensemble-based algorithms—RF and GB—had the highest cumulative performance scores (approximately 4.2 and 4.0, respectively). LR achieved a comparable total score, while DT and MLP contributed slightly lower sums (~3.8–3.9). These results confirm that ensemble learning approaches provided the most reliable and balanced predictions for distinguishing between subclinical and clinical disease states in dairy cows.

### 3.2 Statistical evaluation of physiological and milk composition traits

Significant differences among health status groups were observed for several milk and behavioral parameters. The statistical analysis revealed clear group effects, particularly for FPR, RT, and SCC (*p* < 0.05). These results indicate distinct metabolic and udder health patterns between clinically healthy cows and those with subclinical or clinical disorders.

*Post hoc* comparisons (Table 2) confirmed that cows with subclinical ketosis had significantly higher fat content (mean = 5.21 ± 0.72%) compared with clinically healthy (4.45 ± 0.54%), subclinical mastitis (4.47 ± 0.73%), and clinical mastitis (3.90 ± 0.81%) groups (*p* < 0.001). Lactose content was significantly lower in cows with clinical mastitis (4.56 ± 0.24%) than in clinically healthy cows (4.63 ± 0.16%) (*p* < 0.05). RT differed markedly among all groups: it was longest in clinically healthy cows (477.0 ± 36.0 min/day), intermediate in subclinical mastitis (398.1 ± 72.9 min/day) and clinical mastitis (393.3 ± 83.6 min/day), and shortest in subclinical ketosis (336.9 ± 94.2 min/day) (*p* < 0.001). SCC was dramatically elevated in cows with clinical mastitis (416.8 × 10^3^ cells/mL) compared with all other groups (range 62.5–64.0 × 10^3^ cells/mL) (*p* < 0.001).

**Table 2:** Post hoc (Games–Howell) pairwise group comparisons.

Variable	Comparison (groups)	Mean difference	*p*-value	Interpretation
Fat (%)	2–1	+0.76	<0.001	Higher in subclinical ketosis
Fat (%)	2–3	+0.74	<0.001	Higher in subclinical ketosis
Fat (%)	3–4	+1.31	<0.001	Higher in subclinical ketosis
Lactose (%)	4–1	−0.07	0.030	Lower in clinical mastitis
RT (min/day)	2–1	−140.0	<0.001	Shorter in subclinical ketosis
RT (min/day)	4–1	−83.7	<0.001	Shorter in clinical mastitis
SCC (×10^3^/mL)	4–1	+352.7	<0.001	Higher in clinical mastitis
SCC (×10^3^/mL)	4–2	+354.3	<0.001	Higher in clinical mastitis

Levene’s test revealed unequal variances for fat (*p* = 0.006), protein (*p* = 0.023), rumination time (*p* = 2.54 × 10^−11^), and SCC (*p* = 1.64 × 10^−8^), while variances were homogeneous for lactose (*p* = 0.184) and the fat-to-protein ratio (*p* = 0.197) (Supplementary Table S1). Despite this heterogeneity, the ANOVA indicated clear group-related differences for several traits. Significant effects of health status were observed for fat (*F* = 21.54, *p* = 0.00002), lactose (*F* = 6.55, *p* = 0.0003), rumination time (*F* = 49.91, *p* < 0.001), and SCC (*F* = 50.60, *p* < 0.001), whereas protein (*F* = 0.23, *p* = 0.878) and FPR (*F* = 0.40, *p* = 0.754) (Supplementary Table S2). FPR was used for group classification, but no significant difference was found among groups.

## 4 Discussion

The purpose of this study was to assess the effectiveness of five supervised ML algorithms for the early identification and categorization of metabolic and udder health disorders in dairy cows during the early stages of lactation: Decision Tree, Logistic Regression, Multilayer Perceptron, Random Forest, and Gradient Boosting. Two commercial dairy herds’ automatically gathered data on milk content, rumination duration, and SCC were used to train and validate the models. All models achieved satisfactory classification accuracy, ranging from 0.81 to 0.85, according to the results. These results supported the original premise that, employing multisource sensor data, ensemble-based ML algorithms may successfully distinguish between healthy cows and those with subclinical ketosis, subclinical mastitis, or mastitis.

Based on automatically collected sensor data, the Random Forest model demonstrated superior performance compared to the other tested algorithms and confirmed its strong ability to distinguish between clinically healthy cows and those affected by metabolic or udder health disorders, achieving the highest overall classification accuracy in this study (0.857). Numerous agricultural applications have demonstrated the excellent efficacy of the RF algorithm, an ensemble learning technique intended for classification and regression tasks. The ability of RF to handle complex datasets and support real-time monitoring within integrated sensor-based systems has been highlighted by studies on smart livestock management, which have shown that RF can accurately predict health conditions like milk fever and mastitis by analyzing multiple physiological and production indicators (15). Furthermore, studies have demonstrated that RF can anticipate cattle health problems associated with behavioral and physiological indicators taken from real-time monitoring systems with a high degree of accuracy (16). In a similar vein, our study’s GB and LR models both attained a classification accuracy of 0.833, indicating dependable performance in identifying early metabolic and udder health issues in dairy cows during early lactation. The use of GB to forecast cattle diseases, especially mastitis, highlights the technique’s advantages in managing big datasets with intricate relationships. As an example, Guo et al. (17) evaluated the effectiveness of several models for predicting mastitis and discovered that XGBoost, a particular GB implementation, performed better than other models, such as LR, with an area under the ROC curve (AUC) of 0.75. LR offers simple interpretability, which is crucial for veterinary professionals hoping to extract useful insights from model outputs, whereas GB models frequently offer improved accuracy and are well suited for handling big and complicated datasets. Combining the two methods can be beneficial for thorough health monitoring in dairy herds, enabling GB to improve predictions for more accurate disease diagnosis and LR to function as a transparent preliminary evaluation tool (17, 18). Additionally, the complimentary capabilities of these two approaches are what make them potentially synergistic. It is feasible to create more focused and efficient herd health management techniques that maximize both prediction accuracy and practical applicability by using GB to analyze complicated, multivariate data and LR to assess established risk variables (19).

The DT and MLP models in our study had a good ability to forecast health issues in dairy cows, although their total classification accuracy was the lowest among the tested algorithms, each reaching 0.810. Additionally, by examining physiological and behavioral data gathered from AMS, such as milk yield, RT, and changes in body weight around calving, DT models have shown promise in predicting metabolic and udder health concerns in dairy cows. Strong predictive accuracy in detecting cows at risk of mastitis and ketosis has been shown by these models, underscoring their potential utility for real-time health monitoring and dairy management decision-making (20). Metabolic markers, such as milk fatty acid profiles, have been found to be especially significant in the context of predicting subclinical ketosis because MLP models can accurately capture their nonlinear correlations with ketone body concentrations. MLP-based techniques can improve the precision of subclinical ketosis prediction by utilizing these intricate data patterns, facilitating early detection and intervention (21). Furthermore, MLPs are useful instruments for simulating intricate physiological relationships because of their great nonlinear mapping capabilities, which enhances predictive reliability when examining multifactorial health data in dairy cows (22). Additionally, according to Pala and Çamurcu (23), choosing the right ML approaches is crucial for efficient health management. They talk about how using several algorithms, such as DT and MLPs, might improve health monitoring tactics in dairy herds by producing better prediction outcomes than using them separately.

In our investigation, cows in subclinical ketosis had the shortest RT (336.9 ± 94.2 min/day) and a considerably greater milk fat content (5.21 ± 0.72%), indicating a marked negative energy balance and decreased feed intake. Clinically healthy cows, on the other hand, had the longest RT (477.0 ± 36.0 min/day) and lower fat levels (4.45 ± 0.54%), indicating normal feeding behavior and sustained metabolic activity. Mastitis-affected cows had the lowest milk fat content (3.90 ± 0.81%) and the longest ruminating time (393.3 ± 83.6 min/day), indicating that inflammatory processes may also limit rumination, albeit less so than metabolic diseases. According to earlier studies, cows in ketosis have higher blood levels of NEFA, which act as building blocks for the production of milk fat and raise the amount of milk fat (24). Around the outset of the disease, this metabolic imbalance is frequently accompanied by a significant drop in rumination activity, suggesting a clear link between reduced food behavior and altered metabolic performance. These results imply that while milk fat levels increase during ketosis, the general health and energy status of cows decline, as evidenced by shorter RT (25). Rumination patterns and milk composition, especially fat content, are important indicators of the health of dairy cows. Research indicates that RT reductions frequently take place a few days prior to the clinical diagnosis of mastitis, which is consistent with behavioral alterations seen in cows with subclinical ketosis (26). The importance of these characteristics for predictive modeling and early detection in dairy herd management is highlighted by the fact that disruptions in regular daily activities act as early markers of health decline in both situations. Furthermore, according to Antanaitis et al. (27), even subclinical mastitis has an impact on milk supply and composition and causes notable alterations in rumination behavior. According to their findings, the average ruminating duration for cows with subclinical mastitis decreases by more than 5% during health disturbances, supporting the connection between rumination and health state. Clinical evaluations have shown that these behavioral abnormalities are frequently associated by variations in milk fat content.

In comparison to clinically healthy cows (64.0 × 10^3^ cells/mL and 4.63 ± 0.16%, respectively), cows in the clinical mastitis group showed a significantly higher SCC (416.8 × 10^3^ cells/mL) and a significantly lower lactose content (4.56 ± 0.24%), suggesting an active inflammatory response in the mammary gland and decreased lactose synthesis linked to infection. There is a clear link between mastitis and higher SCC in milk, according to numerous research. For example, SCC levels in cows with mastitis have been shown to be much higher than normal, frequently surpassing 200,000 cells/mL when pathology is present (28, 29). Research demonstrates the importance of subclinical mastitis as an early marker of intramammary infection by confirming that it can cause significant increases in SCC even in the absence of obvious symptoms (29, 30). Furthermore, it is impossible to overstate how mastitis affects milk supply and quality. Reduced milk yield and quality are correlated with an increase in SCC, which also suggests a compromise in udder health (31). For instance, elevated SCC levels are linked to modifications in milk composition, such as a decreased ability to synthesize lactose, which frequently coexists with the milk’s compromised nutritional profile (30). Increases in SCC are indicative of an inflammatory response that disrupts normal milk synthesis, as demonstrated by the well-established correlation between elevated SCC and decreased lactose content. Lactose levels often decrease proportionately as SCC increases in response to mastitis, suggesting compromised mammary epithelial function (32). Additionally, there is evidence that maintaining lactose content and overall milk quality can be achieved by effectively controlling SCC through better herd health management (33).

A number of limitations should be noted, even if the study’s findings show how supervised ML models can be used to detect metabolic and udder health issues in dairy cows using automatically recorded milk and behavioral data. Because illness occurrence and model performance might be influenced by herd-specific management techniques, environmental circumstances, and dietary strategies, the very limited dataset and inclusion of only two herds may limit the findings’ generalizability. Notwithstanding these limitations, the results demonstrate the usefulness of combining ML and multi-sensor data to improve early health detection, allowing for more accurate and proactive herd management. Therefore, future research should include benchmarking against traditional diagnostic criteria, assess the reliability of forecasts under various production systems, and validate these models over bigger and more varied dairy populations. As continuous and non-invasive monitoring could lower labor inputs, minimize animal stress, and enhance the long-term sustainability of dairy production, additional research should take into account the economic and welfare implications of implementing sensor-based ML technologies in addition to diagnostic accuracy. Although ROC and AUC analyses were not included in this study due to its multiclass design, future research focusing on individual disease categories could incorporate these metrics to further evaluate model discrimination performance.

## 5 Conclusion

Based on automatically recorded behavioral and milk composition indicators, this study showed that supervised machine learning algorithms may successfully identify early-stage metabolic and udder health issues in dairy cows. Ensemble algorithms like RF and GB demonstrated the strongest classification performance among the investigated models, correctly differentiating healthy cows from those with mastitis-related illnesses or subclinical ketosis. Additionally, LR had a promising predictive capacity, demonstrating its promise as an understandable and transparent diagnostic tool. DT and MLP models, on the other hand, produced somewhat lower but still respectable classification accuracies, suggesting that even more basic ML architectures can produce useful diagnostic insights when used with precise monitoring data.

Automated rumination tracking devices and in-line milk analyzers have proven very useful in recording subtle physiological and behavioral changes that occur before to the clinical presentation of disease. While elevated SCC and decreased lactose levels were substantially linked to clinical mastitis, increased milk fat content and decreased RT were found to be reliable markers of subclinical ketosis. These findings highlight the diagnostic utility of integrating behavioral measurements with milk composition to facilitate early, non-invasive health evaluation in dairy herds.

The study’s overall conclusions support the use of ML-based techniques in precision cattle husbandry. These technologies can help diagnose metabolic and inflammatory problems early, make better judgments about herd management, and increase animal welfare and production by offering continuous, automated health monitoring. Future research should concentrate on integrating these models into useful on-farm decision-support systems and verifying them across bigger and more diverse dairy populations. Implementing ML-based monitoring systems can significantly improve farm productivity and animal welfare from a wider standpoint. Early and automated diagnosis of health abnormalities minimizes animal discomfort and treatment-related stress by reducing the necessity for invasive diagnostic procedures and the danger of serious clinical disease. Practically speaking, these systems let farm managers to minimize financial losses brought on by lower milk yield or culling, make fast, data-driven choices, and maximize resource utilization. By increasing overall herd lifetime and productivity, the use of ML methods in herd management may help promote sustainable cattle production.

## Data Availability

The original contributions presented in the study are included in the article/Supplementary material, further inquiries can be directed to the corresponding author.
