# Supplementary material for: Supervised machine learning approaches for early detection of metabolic and udder health disorders in dairy cows using sensor-derived data
Source: Front Vet Sci. 2025 Nov 19;12:1726719. doi: 10.3389/fvets.2025.1726719 (PMC12673267; doi:10.3389/fvets.2025.1726719)
Supplement: Supplementary file 1 [file Table_1.docx]

| **Variable** | **F-value** | **df₁** | **df₂** | | **p-value** | | **Interpretation** |
| --- | --- | --- | --- | --- | --- | --- | --- |
| Fat (%) | 4.2738 | 3 | | 202 | | 0.0060 | Variances unequal (*p* < 0.05) |
| Protein (%) | 3.2377 | 3 | | 202 | | 0.0232 | Variances unequal (*p* < 0.05) |
| Lactose (%) | 1.6264 | 3 | | 202 | | 0.1844 | Variances equal |
| RT (min/day) | 19.8595 | 3 | | 202 | | 2.54 × 10⁻¹¹ | Variances unequal (*p* < 0.001) |
| FPR | 1.5716 | 3 | | 202 | | 0.1974 | Variances equal |
| SCC (×10³/mL) | 14.3491 | 3 | | 202 | | 1.64 × 10⁻⁸ | Variances unequal (*p* < 0.001) |
|  |  |  |  |  |  |  |  |

Table 1. Levene’s test for equality of variances among groups.
