# Supplementary material for: Supervised machine learning approaches for early detection of metabolic and udder health disorders in dairy cows using sensor-derived data
Source: Front Vet Sci. 2025 Nov 19;12:1726719. doi: 10.3389/fvets.2025.1726719 (PMC12673267; doi:10.3389/fvets.2025.1726719)
Supplement: Supplementary file 2 [file Table_2.docx]

| **Variable** | **F-value** | **df (between, within)** | **p-value** | **Interpretation** |
| --- | --- | --- | --- | --- |
| Fat (%) | 21.540 | (3, 202) | 0.0002 | Significant difference (*p* < 0.001) |
| Protein (%) | 0.230 | (3, 202) | 0.878 | Not significant |
| Lactose (%) | 6.550 | (3, 202) | 0.0003 | Significant difference (*p* < 0.01) |
| RT (min/day) | 49.910 | (3, 202) | <0.001 | Significant difference (*p* < 0.001) |
| F:P | 0.400 | (3, 202) | 0.754 | Not significant |
| SCC(×10³/mL) | 50.600 | (3, 202) | <0.001 | Significant difference (*p* < 0.001) |

Table 2. One-way ANOVA results for milk and physiological parameters among groups.
